# Prosocial behavior in children involved in peer violence

**DOI:** 10.47626/2237-6089-2023-0632

**Published:** 2024-10-28

**Authors:** Marília M. Mendes-Sousa, Anderson Ribeiro da Silva, Marília Mariano, Rosângela Espolaor, Raquel Fernandes Shimizu, Jair J. Mari, Zila M. Sanchez, Sheila C. Caetano

**Affiliations:** 1 Universidade Federal de São Paulo Departamento de Psiquiatria e Psicologia Médica São Paulo SP Brazil Departamento de Psiquiatria e Psicologia Médica, Universidade Federal de São Paulo (UNIFESP), São Paulo, SP, Brazil.; 2 Área de Epidemiologia e Bioestatística Departamento de Medicina Preventiva São Paulo SP Brazil Departamento de Medicina Preventiva, Área de Epidemiologia e Bioestatística, UNIFESP), São Paulo, SP, Brazil.

**Keywords:** School violence, peer violence, prosocial behavior, disruptive behavior

## Abstract

**Objective:**

Peer violence is a serious type of school violence that is associated with emotional and behavioral problems. The objective of this study was to analyze violence between peers and its associations with students’ social skills.

**Methods:**

We used a cross-sectional survey nested within a cluster-randomized controlled trial (REBEC/Brazil, U1111-1228-2342) to evaluate peer violence among elementary school students and its association with prosocial behaviors and mental problems. Teachers answered an adapted version of the Revised Olweus Bully/Victim Questionnaire (OBVQ) and the Brazilian adaptation of the Teacher Observation of Classroom Adaptation-Checklist (TOCA-C) scale for each student. Children completed a sociodemographic questionnaire. The participants were 1,152 5-to-14-year-old children from Brazilian public schools, 79.70% of whom reported being involved in violent situations.

**Results:**

Children who had both committed and suffered violence were less likely to exhibit prosocial behaviors. Children who committed and suffered violence and those who only committed violence were more likely to experience concentration problems and exhibit disruptive behaviors.

**Conclusion:**

This study suggests that peer violence is associated with fewer prosocial behaviors and more behavioral problems. Thus, more specialized mental health care is required for children involved in peer violence, in addition to implementation and maintenance of programs to prevent and reduce violence and develop prosocial behaviors in schools.

## Introduction

Peer violence happens frequently in school settings. Such behavior is characterized by aggressive incidents between peers, aiming to hurt the victim through vexatious and embarrassing situations. The forms of violence are physical, psychological, and moral, such as kicking, hitting, badmouthing, spreading rumors or gossip, persuading and manipulating others to stop talking to the victim, and stealing their belongings, among other aversive behaviors. When these phenomena happen repeated times and with lasting effects, they can be considered bullying.^1–3^ In this study, we have used the term "peer violence," because the frequency of violence among students was not verified.

In 2018, the Programme for International Student Assessment (PISA) conducted a survey of 79 countries, finding that 29% of adolescents in Brazil reported being bullied a "few times a month"; the mean in other countries was 23%.^4^ The Teaching and Learning International Survey (TALIS), conducted in 48 countries in 2018 by the Organization for Economic Cooperation and Development (OECD), reported that in Brazil, 28% of principals working with the final years of elementary education reported daily or weekly bullying among students, compared to the Latin American average of 13%, showing that Brazil has higher rates of bullying in school environments than other countries.^5,6^

Children and adolescents exposed to an active peer violence context may present associated psychological and psychiatric problems.^7^ Since child development is a vulnerable process, there is a greater chance of initiating deviant attitudes during this phase and maintaining violent behavior over time, such as bullying.^8–10^ The negative effects of peer violence on mental health can lead to inappropriate behaviors with oneself or others, such as problems inside and outside the school setting, substance abuse, violation of norms and laws, behavioral and emotional problems, and a lack of social skills, including affective and educational skills, during school and after coming of age.^3,11–13^ However, child mental health problems can also contribute to peer violence.^14^

Construction of social skill repertoires is associated with the subject's cultural context; thus, behaviors associated with assertiveness in communication, opinions, and attitude may be viewed as prosocial behaviors under the aegis of stabilized social norms.^15^ Decreased or absent social skills, which are essential protective factors against behavioral problems, can be perceived in those who commit violence, suffer violence, and both commit and suffer violence.^16^

Prosocial behaviors, characterized through social skills, are defined by altruistic processes and positive acts with the intention of promoting the well-being of others. Furthermore, these behaviors are defined as interpersonal processes aimed at voluntarily benefiting others.^17–19^ That is, being prosocial improves friendship development.^20^ A lack of social skills can be characterized by reinforcement of behaviors that hinder socialization, such as disruptive, aggressive, or repressive behaviors, or even classroom difficulties, concentration problems, and low academic performance. These behaviors, however socially undesirable in different contexts, such as school, may be adaptive for the individual, as protective mechanisms.^21^

Most studies of the subject in the literature have evaluated high-income countries, showing how the mental health needs of children from low-income countries are neglected, becoming major public health concerns.^22,23^ It is thus hypothesized that children who commit and suffer violence are less likely to present prosocial behaviors and more likely to present mental health problems related to concentration problems and disruptive behaviors. The objective of this study was to analyze peer violence and its associations with these specific behavioral repertoires in public elementary school students in Brazil.

## Methods

### Participants and environment

This is a cross-sectional study nested in a randomized controlled trial that evaluated the effectiveness of the Elos 2.0 Program targeting reduction of problem behaviors and promotion of social skills in schoolchildren (REBEC/Brazil, U1111-1228-2342, https://ensaiosclinicos.gov.br/rg/RBR-86c6jp).^24^ This study used only the baseline data collected in 2019. It included 1,152 children aged from 5 to 14 years, enrolled in the first to fourth grades of elementary school at 11 public schools in the city of Fortaleza and the town of Eusébio (Ceará, Brazil) and their teachers (n = 40).

### Data collection procedures

University students on health-related courses were trained to administer the instruments to students and teachers. The instrument was individually administered to the teachers in a place that enabled confidentiality. The students were aided by field research assistants who read the questions aloud in the classroom and showed where the "yes" and "no" options for each question would be in the questionnaire.

### Ethical considerations

The research project was approved by the ethics committee at the Universidade Federal de São Paulo (CAAE: 01517218.2.0000.5505, n: 1246/2018). The informed consent form ensured subjects’ participation was voluntary, explained the research objectives and how the information would be used, and made volunteers aware of the procedures to which they would be subjected and their possible consequences.

### Instruments and variables

The children's instrument to assess peer violence was an adapted version of the Olweus Bully/Victim Questionnaire (OBVQ).^10,25^ The original questionnaire measures bullying (23 items) and being bullied (23 items) in the previous 30 days. Responses are given on a 1-4 Likert scale ranging from "never" to "several times a week." Each item describes a behavior and the frequency with which it occurred, such as: "said mean things about him/her or about his/her family" (aggressor's version) or "made or tried to make other students dislike me" (victim's version). Due to children's difficulty with responding using a Likert scale, the response options were changed to "yes" or "no," thereby misrepresenting the measurement factor, so it was not possible to analyze the frequency with which violence occurred, but nevertheless maintained the construct of violent/aggressive behaviors. Violent behaviors were divided into exclusive categories: "committed and suffered violence," "only committed," "only suffered," and "did not commit or suffer violence." Data on the children's gender and age were also collected for model adjustment. Previous research shows that use of this instrument is effective for studies with younger children.^26,27^

The teachers responded to the Teacher Observation of Classroom Adaptation-Checklist (TOCA-C), which assesses the behavior of each student in the classroom^28^ and has been adapted for the Brazilian context.^29^ The adapted instrument has 21 items answered on a three-point scale ("rarely," "sometimes," and "frequently"); and the factors assessed were grouped into prosocial behavior, disruptive behavior, and concentration problems. A high score on the prosocial factor indicates positive behaviors, whereas higher scores on the other two factors indicate negative behaviors.

The teachers’ sociodemographic questionnaire included information on gender, age, and education. Socioeconomic level was assessed using the Associação Brasileira de Empresas de Pesquisa (ABEP)^30^ index, which is based on the educational level of the head of the family, ownership of several consumer goods, and the number of household employees. In this classification, groups A and E are the highest and lowest classes, respectively.

### Statistical analysis

We presented descriptive characteristics for the child and teacher samples. For categorical variables, absolute and relative frequencies were reported; for numerical variables, summary measures (mean and standard error) were presented.

We then performed a multinomial logistic regression in which the outcomes were the children's social skills, disruptive behaviors, and concentration problems (measured by the TOCA-C and classified without overlapping categories) and the exposure was "violent/aggressive behaviors." The regression was controlled for the children's gender and age. A 95% confidence interval (95%CI) and a significance level of p < 0.05 were adopted. Stata Statistical Software version 15 was used for the analyses.

## Results

Table 1 presents the descriptive data of students and teachers. The prevalence of children's involvement in peer violence in this study was 79.70%, with 7.03% having committed some form of peer school violence, 25.23% having experienced violence, and 47.45% having engaged in or experienced both in the previous month. The socioeconomic status listed in the table is the teachers’. The students are children from public schools, middle and lower class, who cannot afford private schools.

**Table 1:** Sociodemographic characteristics of students and teachers and teachers’ employment characteristics

Variable			n	w% or mean	w95%CI
Student variables (n = 1,112)					
	Gender				
		Male	552	49.6	(0.47-0.53)
		Female	560	50.4	(0.47-0.53)
	City				
		Capital	934	84.0	(0.82-0.86)
		Metropolitan region	178	16.0	(0.14-0.18)
	School year				
		1st	188	16.9	(0.15-0.19)
		2nd	205	18.4	(0.16-0.21)
		3rd	261	23.5	(0.21-0.26)
		4th	458	41.2	(0.38-0.44)
	Period				
		Morning	714	64.2	(0.61-0.67)
		Afternoon	398	35.8	(0.33-0.39)
	Age				
		5	19	1.7	(0.01-0.02)
		6	188	16.9	(0.15-0.19)
		7	231	20.8	(0.18-0.23)
		8	263	23.7	(0.21-0.26)
		9	302	27.2	(0.24-0.30)
		10	71	6.4	(0.05-0.08)
		11	26	2.3	(0.01-0.03)
		12	7	0.6	(0.00-0.01)
		13	3	0.3	(0.00-0.01)
		14	2	0.2	(0.00-0.00)
					
Teacher variables (n = 40)					
	Educational level				
		Complete high school or incomplete undergraduate degree	1	2.5	(-0.02-0.07)
		Complete undergraduate degree to incomplete graduate degree	17	42.5	(0.27-0.58)
		Complete graduate degree	22	55.0	(0.40-0.70)
	Time working as a teacher (years)				
		0-4	5	12.5	(0.02-0.23)
		5-9	6	15.0	(0.04-0.26)
		10-14	5	12.5	(0.02-0.23)
		15 >	24	60.0	(0.45-0.75)
	Number of schools at which teachers work				
		1	33	82.5	(0.71-0.94)
		2	6	15.0	(0.04-0.26)
		3	1	2.5	(-0.02-0.07)
	Number of classes currently taught				
		1	4	10.0	(0.01-0.19)
		2	28	70.0	(0.56-0.84)
		3	6	15.0	(0.04-0.26)
		4	2	5.0	(-0.02-0.12)
	Working hours				
		30	1	2.5	(-0.02-0.07)
		40	38	95.0	(0.88-1.02)
		> 40	1	2.5	(-0.02-0.07)
	Socioeconomic status*				
		B2	14	35.0	(0.20-0.50)
		B1	6	15.0	(0.04-0.26)

95%CI = 95% confidence interval; B = middle high; w = weighted.

* According to the Associação Brasileira de Empresas de Pesquisa (ABEP) clasification.^28^

Table 2 presents the unadjusted multinomial logistic regression conducted to analyze the outcomes prosocial behaviors, disruptive behaviors, and concentration problems in children exposed to violent social interactions or aggressive behaviors reported by students in the view of teachers. Table 3 shows the multinomial logistic regression adjusted for age and gender considering the outcomes prosocial behaviors, disruptive behaviors, and concentration problems, with peer violence as exposure. The results of the adjusted and unadjusted models are extremely similar, suggesting that we should only report the adjusted results.

**Table 2:** Unadjusted multinomial logistic regression with prosocial, disruptive, and concentration behaviors as outcomes and peer violence as exposure

		Peer violence - Not involved (ref.)					
		Committed and suffered peer violence		Suffered peer violence		Committed peer violence	
		RRR	95%CI	RRR	95%CI	RRR	95%CI
Prosocial (frequently ref.)							
	Rarely	**2.515**	**1.481-4.268**	1.456	0.797-2.662	0.934	0.336-2.594
	Sometimes	**2.159**	**1.447-3.157**	0.997	0.632-1.574	**2.069**	**1.174-3.645**
							
Disruptive behaviors (rarely ref.)							
	Sometimes	**3.008**	**1.634-5.553**	1.683	0.839-3.374	**2.835**	**1.192-6.738**
	Frequently	**2.353**	**1.472-3.762**	1.841	0.866-2.531	**2.457**	**1.237-4.876**
							
Concentration problems (rarely ref.)							
	Sometimes	**2.670**	**1.810-3.937**	1.465	0.943-2.278	**2.021**	**1.100-3.714**
	Frequently	**2.790**	**1.924-4.046**	1.413	0.923-2.162	**2.195**	**1.237-3.893**

95%CI = 95% confidence interval; RRR = relative risk reduction, ref. = reference category.

Bold text indicates statistically significant odds ratios at p < 0.05.

**Table 3:** Multinomial logistic regression adjusted for age and gender with prosocial, disruptive, and concentration behaviors as outcome and peer violence as exposure

		Peer violence - Not involved (ref.)								Gender – Boy (ref.)	
		Committed and suffered peer violence		Suffered peer violence		Committed peer violence			Age		Girl
		RRR	95%CI	RRR	95%CI	RRR	95%CI	RRR	95%CI	RRR	95%CI
Prosocial (frequently ref.)											
	Rarely	**2.419**	**1.419-4.126**	1.451	0.791-2.660	0.838	0.300-2.342	0.903	0.792-1.031	**0.489**	**0.337-0.709**
	Sometimes	**2.393**	**1.622-0.530**	1.046	0.658-1.663	**2.152**	**1.202-3.853**	**0.652**	**0.586-0.727**	0.963	0.731-1.268
											
Disruptive behaviors (rarely ref.)											
	Sometimes	**2.927**	**1.587-5.398**	1.665	0.829-3.345	**2.564**	**1.073-6.126**	0.935	0.813-1.076	**0.591**	**0.401-0.870**
	Frequently	**2.098**	**1.304-3.374**	1.385	0.806-2.381	**2.178**	**1.084-4.374**	**1.323**	**1.184-1.479**	**0.573**	**0.414-0.792**
											
Concentration problems (rarely ref.)											
	Sometimes	**2.766**	**1.868-4.096**	1.508	0.967-2.352	**1.972**	**1.065-3.651**	**0.770**	**0.694-0.855**	0.802	0.610-1.054
	Frequently	**2.550**	**1.751-3.714**	1.359	0.885-2.089	**1.953**	**1.093-3.488**	**1.168**	**1.064-1.283**	**0.542**	**0.417-0.706**

95%CI = 95% confidence interval; RRR = relative risk reduction, ref. = reference category.

Bold text indicates statistically significant odds ratios at p < 0.05.

Table 3 shows that, regarding prosocial behaviors, children who committed and suffered violence have a higher adjusted odds ratio (aOR) of presenting fewer prosocial behaviors than children who presented many prosocial behaviors (aOR = 2.42, 95%CI 1.41-4.12, p < 0.05). Median prosocial behaviors scores were significant for children who committed and suffered violence, who committed violence, and who were younger (aOR = 2.39, 95%CI 1.62-3.53; aOR = 2.15, 95%CI 1.20-3.85; aOR = 0.65, 95%CI 0.59-0.73, respectively).

Regarding disruptive behaviors, children who committed and suffered violence and children who only committed violence had a higher aOR of developing such behavior (aOR = 2.93, 95%CI 1.59-5.40; aOR = 2.56, 95%CI 1.07-6.13, respectively) than children who neither committed nor suffered violence. Age was also relevant (aOR = 1.32, 95%CI 1.18-1.48).

Children who committed and suffered peer violence or who only committed violence had a 2.77 (95%CI 1.87-4.10) and 1.97 (95%CI 1.06-3.65) greater chance of having concentration problems, respectively, than children who neither committed nor suffered violence. Additionally, age is a relevant factor with regard to concentration problems, with older children having a 71% (95%CI 1.0-1.28) greater chance at each 1-year increment of having problems related to this behavior.

As for gender, boys are more likely to have more concentration problems and to exhibit more disruptive behaviors and fewer prosocial behaviors than girls: 0.54 (95%CI 0.42-0.71), 0.59 (95%CI 0.4-0.87), and 0.49 (95%CI 0.34-0.71), respectively.

## Discussion

Our hypothesis that elementary school children who have higher rates of peer violence behaviors (committed/suffered) at school are associated with poor social skills, disruptive behaviors, and higher chances of concentration problems was corroborated. In our study, students who committed, and who both committed and suffered peer violence were more likely to present few prosocial behaviors. Researchers who evaluated 39,936 school children aged 7-14 (from Finland, the Netherlands, and the United Kingdom) also found a negative association between prosocial behavior and teacher-rated aggressive behavior. Therefore, the phenomenon of violence seems to be associated with decreased assertive behaviors that are consistent with good peer relationships. Children who committed and suffered peer violence seem to be less adaptive and have lower rates of prosocial behavior, possibly because they are involved in both roles of the phenomenon.^31^

It is important for child development to learn behaviors related to emotional expressiveness, problem solving through assertiveness, good relationships with others, and understanding the rules of the environment. In general, responses aimed at social skill practices produce more reinforcers for children in their collective development environment, helping to reduce risky behaviors such as peer violence.^16,32^ Acquired and improved social skills seem to be predictive of good school performance. These skills are related to child autonomy, caring, and a sense of justice.^33,34^

Disruptive behaviors generate aggressive interactions. In our results, students who committed and who both committed and suffered peer violence exhibited disruptive behaviors across multiple intensity categories. This is in agreement with researchers who reported that disruptive behaviors are twice as common in children who commit violence and three times as common in victims of violence and that the co-occurrence of externalizing behaviors and peer violence has been observed in younger students.^35,36^ Children who both committed and suffered violence had lower ratings for classmate and teacher relations than children who were not involved in violence.^37^ Bullying victimization in adolescents has been extensively documented as a strong predictor for the emergence of internalizing and externalizing problems, but very few studies demonstrated that children who encountered elevated levels of traditional bullying victimization exhibited a greater likelihood of experiencing both internalizing and externalizing problems.^38^ Disruptive behaviors in the victims of violence and children who commit violence may also be associated with other factors such as depression, anxiety, and attention deficit hyperactivity disorder (ADHD).^7,14^ Children who commit peer violence specifically tend to engage in more problematic conduct and increased hyperactivity compared to their peers and children who are not involved in the phenomenon.^37^ It is noteworthy that children who commit violence and are diagnosed with disruptive behaviors present high levels of aggression and a lack of empathy.^39^

In this study, girls encountered a greater degree of emotional distress related to violent behaviors. Nevertheless, their response to this phenomenon exhibited a more prosocial nature when compared to boys.^40^ Moreover, some children who commit this type of violence are esteemed by their peers, either for affection or admiration, showing themselves to be popular and proactive, thereby acknowledging that the aggressions committed are barely recognized as aversive behavior.^41^ Being a girl stands out in this respect as girls develop greater communication and socialization skills.^42^

Girls also have fewer concentration problems and disruptive behaviors linked to violent relationships, regardless of whether they perform the role of aggressor or victim. Other studies have shown that boys are more involved in all types of violence than girls, with the exception of spreading rumors and gossiping.^9,43^ Aggressive physical interaction is a dominant peer violence practice primarily perpetrated by boys.^44,45^ Boys involved in this type of violence have lower school grades than their peers. Peer rejection appears to result in development of an antisocial attitude over a number of years, such as aggressive, oppositional, and disruptive behaviors.^46^

Regarding the age of the children, this study indicates that younger students tend to exhibit prosocial behaviors more frequently than older students, which reinforces the need to create preventive programs that continuously work to reduce risk factors such as violent social interactions or growing up in a threatening environment, and to enhance protective factors.^47^

Data has shown that teachers are working increasingly longer hours and spending more years in the profession.^48^ While experience may enhance communication skills with students, specialized training is crucial for establishing stronger connections and fostering advanced communication abilities. The primary aim is to encourage positive behaviors and address disruptive ones effectively. Notably, a recent review has shown that teachers’ preparedness plays a crucial role in their involvement following bullying incidents. Schools that implement whole-school antibullying programs and offer training to enhance staff efficacy demonstrate more proactive responses to such situations.^49^ Additionally, it is essential for educational systems to provide support mechanisms that help teachers manage the long-term effects of job stress and prevent burnout, thus maintaining their mental well-being and effectiveness throughout their careers.^50^

School violence in children is a global public health problem. Schools aim to nurture citizens within an institution that should be considered safe for the physical, psychological, and social development of children and adolescents. Understanding the causes of peer violence requires exploration of various theoretical frameworks, including system-level frameworks (e.g., social-ecological, family environment, and relationships within school) and individual-level frameworks (e.g., genetics, developmental psychopathology).^47^ In general, aggressive relationships within educational settings can lead to dominant behaviors that result in severe consequences. These include difficulties in adhering to educational processes, increased risks of school dropout, and significant psychological, physical, and material damages. Specifically, ‘material damage’ relates to the theft or intentional damage of other students’ belongings, while ‘physical damage’ refers to directly inflicting harm on other students. Both types of damage severely impact students’ well-being and educational experiences, as evidenced by the bullying behavior scale we utilized.^2,3^ The involvement of children in peer aggression can generate social rejection from both children of the same age and from teachers and staff.^40^ Violent interactions seem to affect the personalities and self-confidence of children and, as a result, students lose interest in learning and the possibility of inserting themselves in assertive groups; moreover, they feel too intimidated to attend school and focus on their academic activities because they feel emotionally unprepared or need to constantly dedicate themselves to avoiding violence or, conversely, exacting revenge.^44,45^

This study has some limitations. Social desirability may have influenced the responses to the instrument. The teachers often responded during class breaks, in noisy break rooms, and in the presence of other professionals. The students responded to the instrument during class, where the environment may have influenced their responses in some way, such as due to noise and climate. Furthermore, the student data collected from the teachers may present biases since the children did not answer the questions themselves, or because data were not directly observed in the classroom.

The TOCA-C instrument is an excellent low-cost tool that is not time-consuming. It uses data from teachers who interact with children on a daily basis. A longitudinal study is recommended to investigate causality, with assessments answered by students and direct observations.

Peer violence is a significant global public health concern among children and adolescents. Research indicates that both committing and suffering peer violence are linked to current and future mental health issues. Effective intervention strategies should encompass multiple systems and cater to the specific needs of individuals involved in peer violence, ultimately reducing the impact of this risk factor on mental health.^51^ This study underscores the importance of implementing targeted measures to address mental health concerns within educational settings, including prevention programs, psychosocial monitoring of children in development, and communication initiatives aimed at bolstering the crucial foundations of childhood: the school and family environments. In light of the findings, several aspects deserve attention in future research. It is important to highlight the family as a fundamental network in the development of children's behaviors, whether prosocial or disruptive, with transmission of values and family relationships being valuable topics for future investigations and, furthermore, for promoting implementation of prevention programs that actively involve this pillar. Additionally, it is imperative to probe the presence of mental health issues and social cognition performance in individuals exhibiting disruptive behaviors, concentration difficulties, and limited prosocial tendencies. An in-depth analysis of the interplay between these aspects should lead to a more comprehensive understanding of their collective impact on individuals’ lives. These interconnected factors have significant implications for the overall well-being and healthy development of children and adults, encompassing family members, educational professionals, and the children themselves in the long term. To this end, further examination of these relationships in forthcoming research could be pivotal for identifying effective interventions and augmenting existing support systems. Ultimately, such endeavors have the potential to cultivate an environment conducive to fostering healthy socioemotional growth.

In summary, this study suggests that peer violence, regardless of whether it is associated or not with suffered violence, is associated with lower levels of prosocial behaviors and more concentration problems and disruptive behaviors. Thus, more specialized mental health care should be provided to children involved in peer violence. Furthermore, it is important to report that, even though the definition of bullying was not used, this study is relevant because of the age group studied, enabling preventive actions and interventions to ensure that such peer violence does not develop into more extreme situations throughout childhood and adolescence.
